# The effect of a seven-week online mindfulness intervention on the relationship between menstrual symptoms and mental health - A non-randomized controlled trial

**DOI:** 10.1186/s40359-026-05888-2

**Published:** 2026-11-04

**Authors:** Lisa Kullik, Tim Birnkraut, Michael Kellmann, Sarah Jakowski

**Affiliations:** 1 Department of Sport Psychology, Faculty of Sport Science, Ruhr University Bochum, Bochum, Germany; 2 School of Human Movement and Nutrition Sciences, The University of Queensland, Brisbane, Australia

**Keywords:** Menstrual symptoms, Mental health, Anxiety, Mindfulness, Students

## Abstract

**Background:**

Menstrual symptoms are common among physically active women and may affect their well-being and performance. Moreover, symptom frequency has been associated with mental health in previous studies. Although mindfulness-based interventions (MBI) have shown beneficial effects on psychological and physiological health outcomes, their potential role in the relationship between menstrual and mental health remains unclear. Therefore, this study examined whether participation in an MBI was associated with changes in this relationship.

**Methods:**

39 physically active students were split into an experimental group (EG; *n* = 14; 22.68 ± 2.23 years) and a control group (CG; *n* = 25; 22.76 ± 4.88 years), which conducted a baseline and post-intervention measurement, including a psychometric screening. The CG received no intervention during the study and was offered access to the intervention materials afterwards. Assessed constructs included the 21-item version of the Depression Anxiety Stress Scale and the Menstrual Symptom index (MSi). The MBI was developed based on mindfulness-based stress reduction, incorporating the dimensions of acting with awareness, non-judgmental acceptance, and present-moment attention.

**Results:**

At baseline in the control group, MSi scores were positively associated with anxiety (B = 0.16; *p* = .004) and depression (B = 0.14; *p* = .012). No significant group × time interactions were observed for depression or stress. For anxiety, the group × time interaction was significant at the grand-mean MSi score (B = − 3.30; 95% CI [− 6.50, − 0.11]; *p* = .043), whereas the three-way interaction was not significant (*p* = .054).

**Conclusion:**

The results indicate that menstrual symptom frequency is associated with mental health outcomes in physically active students. Participation in the MBI was associated with changes in anxiety over time. However, the non-significant three-way interaction provided no conclusive evidence of moderation. These preliminary findings suggest potential benefits of MBI for anxiety in menstrual health context. Future studies should investigate programs that explicitly address menstrual-related aspects.

**Trial registration:**

This study was registered as ‘Impact of an online mindfulness intervention on sleep quality in physically active students’ in the German Clinical Trials Register (DRKS) under the identifier DRKS00038462 on 13 November 2025. Retrospectively registered.

**Supplementary Information:**

The online version contains supplementary material available at https://doi.org/10.1186/s40359-026-05888-2 .

## Introduction

Research consistently shows that women are at significantly higher risk than men of developing depressive or anxiety disorders [1]. Anxiety disorders, for example, are characterized by emotional reactions such as fear, worry, and tension in response to real or perceived threats [2]. Corresponding to the typical age range for the onset of mental disorders, the developmental phase of emerging adulthood spans the late teenage years through the early twenties [3]. This also marks the period when young adults enter higher education and athletes move from junior to senior levels, potentially causing additional challenges and stressors. Although exercise is generally beneficial for overall health, physically active students, particularly student-athletes, may face a heightened risk of insufficient recovery and mental health challenges due to the combined pressures of athletic, academic, and personal demands [4–6]. These demands may contribute to the high prevalence of depressive symptoms among collegiate student-athletes, estimated between 15% and 33%, with first-year students and women being particularly vulnerable [4, 6]. Moreover, signs of anxiety remain prevalent among student-athletes, with females more likely to exhibit elevated levels of both state and trait anxiety [6].

Menstrual cycle-related hormonal and symptomatic changes may be particularly relevant to this vulnerability, as Li and Graham [7] proposed that fluctuations in reproductive hormones across the menstrual cycle and lifespan may disrupt emotion regulation and thereby increase susceptibility to anxiety disorders. Variations in sex hormones may alter endogenous anxiolytic systems such as serotonin and allopregnanolone, leading to temporary periods of increased anxiety sensitivity, regardless of external circumstances [7]. In line with this, phase-dependent symptom patterns have been observed, with the menstrual phase showing higher symptom intensity and the premenstrual phase greater physical stress [8]. Regarding psychological and social factors, Jang and Elfenbein [9] reported that menstruation is associated with low self-esteem, anxiety, and dysphoria, as well as behavioural changes like social withdrawal that can contribute to depressive moods. Cultural stigma and restrictive attitudes toward menstruation may further heighten stress and negatively affect mental well-being. Recent findings among Turkish female university students acknowledged this by reporting that a higher frequency of menstrual symptoms was linked to greater social appearance anxiety [10]. Decreased body satisfaction in the late luteal and premenstrual phases may heighten appearance concerns, making body image an additional stressor that amplifies the psychological impact of menstrual symptoms. Overall, approximately one in four adolescents experiences significant menstrual disturbances, such as severe pain and multiple concurrent symptoms, which negatively affect daily functioning, academic participation, and social relationships [11]. Armour et al. [12] reported that 71% of the women in their sample experienced dysmenorrhea, and many of them described academic difficulties, including school absenteeism and reduced concentration. Consequently, many young women frequently miss classes, while those who attend often struggle with decreased academic performance.

The week preceding menstruation has received particular attention in research, as it is characterized by various symptoms and impairments commonly referred to as premenstrual syndrome (PMS) [13]. This phase has been associated with increased vulnerability to anxiety and involves both physical and emotional symptoms that can negatively affect well-being and quality of life [14, 15]. One possible explanation is the association between fluctuations in ovarian hormones and psychological symptoms [9]. However, limited research exists on menstrual-related symptoms experienced across the different phases of the cycle, rather than solely during menstruation and before [16, 17]. Early research by Hart and Russell [18] observed that women with PMS not only experienced stronger premenstrual symptom increases but also showed consistently higher baseline levels of anxiety, tension, and depression across the entire menstrual cycle, suggesting that approaches should target emotional well-being throughout the cycle rather than focusing solely on the premenstrual phase. Kullik et al. [19] also reported a positive association between menstrual symptom frequency and mental health outcomes, with no effects of a specific cycle phase. Moreover, among physically active women, greater general menstrual symptom frequency was significantly linked to missed or adjusted training sessions, absenteeism from competitions or academic/work obligations, and a higher use of pain medication [16].

Given the potential impact of menstrual symptoms on students’ academic and athletic participation, it is essential to explore interventions that can reduce symptom burden and improve coping. A promising approach for managing psychological and physical symptoms associated with the menstrual cycle is mindfulness-based stress reduction (MBSR). Mindfulness is typically described as the intentional and nonjudgmental attention to one’s experiences in the present moment [20]. Both trait and state mindfulness have been associated with positive health outcomes, such as reduced stress, enhanced emotional regulation, and healthier behavioural patterns [21, 22]. These mechanisms may be particularly relevant in the context of menstrual symptoms, as mindful awareness and acceptance may represent protective factors against premenstrual distress and functional impairment [23]. Outcomes, such as depression, anxiety, and stress have shown improved results when treated with mindfulness-based interventions (MBI) [24, 25]. Multiple studies have investigated the effect of MBI on menstrual complaints. Mindfulness appears to moderate the relationship between menstrual attitudes and perceived symptom intensity [15]. For instance, Huang et al. [26] found that MBI significantly increased mindfulness and reduced anxiety symptoms in women with menopausal syndrome, while Salajegheh et al. [27] reported similar benefits for women with polycystic ovary syndrome, including a significant decrease in worry levels. In women with menstrual-related mood disorders, Bluth et al. [28] reported that an eight-week MBI significantly alleviated symptoms including depression, anxiety, mood swings, and irritability. In recent years, several studies, particularly from the Middle East and North Africa region, have reported promising effects of MBI among women with PMS. These include reductions in physical, behavioural, and psychological symptoms [29–31] and improvements in quality of life [31]. Beyond the menstrual health context, Tingaz et al. [32] found that mindfulness was associated with better self-rated athletic performance and lower depression, anxiety, and stress in student athletes. In the study by Mazaheri Asadi et al. [31], the MBI was made accessible to participants via a mobile application and yielded promising results. This supports the perspective of Rice et al. [33], who highlight that digital technologies offer valuable opportunities to implement such interventions, particularly among digital-native populations adept at using computer- and internet-based tools. However, the existing evidence is limited by heterogeneous populations, differences in intervention formats and outcomes, restricting the generalizability of findings to healthy young women. Moreover, to our knowledge, it remains unclear whether an online MBI may moderate the relationship between menstrual symptom frequency and mental health outcomes.

Given the high prevalence of menstrual-related symptoms among young women and their established associations with mental health outcomes, investigating this relationship in the context of an online MBI is particularly relevant, as evidence on accessible non-pharmacological approaches addressing both aspects remains limited. Theoretically, mindfulness is assumed to act less on the occurrence of menstrual cycle-related symptoms than on how they are experienced. Mindfulness has been linked to lower anxiety and depression via emotion regulation processes, particularly less worry and rumination and greater cognitive reappraisal [34]. As cognitive processes such as biased attention to and interpretation of threat contribute to the maintenance of anxiety [35], a present-focused and accepting stance may reduce the extent to which symptoms are appraised as threatening. Although the menstrual-specific evidence outlined above stems from premenstrual symptoms [15, 23], it was assumed that comparable processes apply to symptoms experienced during menstrual bleeding, so that the association between symptom frequency and psychological distress would be weaker after mindfulness training. Based on the existing evidence, we hypothesized that (i) greater menstrual symptom frequency would be associated with higher levels of depression, anxiety, and stress symptoms, and (ii) the online MBI would moderate these associations, such that the positive relationships between symptom frequency and mental health outcomes would be attenuated following the intervention in the experimental group. By testing these hypotheses, the present study contributes to the existing literature by examining self-reported symptom frequency rather than focusing exclusively on clinically diagnosed menstrual disorders in physically active young women in Germany.

## Materials and methods

### Participants

The present sample represents a subset of the overall study population that participated in the intervention, including both male and female participants. The experimental group (EG) consisted of 14 women, and the control group (CG) of 25 women. The descriptive baseline characteristics of the study sample are presented in Table 1. Participants in the EG were recruited through sport psychology courses offered by the Faculty of Sport Science at Ruhr University Bochum. Participants in the CG were recruited among students of the Faculties of Sport Science and Psychology at Ruhr University Bochum. Psychology students were additionally recruited through the faculty’s participant recruitment portal, while further participants were recruited through an online call distributed via student councils at various universities in Germany. All participants could receive study participation credits for their participation in the study. 53.85% (*n* = 21) of the participants reported using hormonal contraception. Naturally menstruating participants (46.15%; *n* = 18) reported a regular menstrual cycle lasting 21–35 days. Eligibility for study participation required a physical activity level of at least 2 sessions per week, each lasting 90 min, corresponding to Tier 1 (Recreationally Active) in the classification by McKay et al. [36]. In total, 28% (*n* = 11) of the participants were classified as Tier 2 (Trained/Developmental), representing the sports soccer, handball, volleyball, basketball, strength training, and climbing. All participants gave written informed consent to participate in the study.

**Table 1:** Baseline demographic and menstrual characteristics of the study sample

	Experimental Group			Control Group			Total		
	(*n* = 14)			(*n* = 25)			(*N* = 39)		
Age	22.68	±	2.23	22.76	±	4.88	22.73	±	4.11
Weekly training duration	7.07	±	4.41	5.17	±	1.87	5.89	±	3.17
Age of Menarche	13.80	±	1.75	12.73	±	1.32	13.06	±	1.52
Cycle Length	27.29	±	2.06	28.70	±	3.86	28.12	±	3.24
Menstruation Length	4.82	±	1.40	5.23	±	1.11	5.09	±	1.21
Heaviness of Bleeding	2.42	±	0.90	3.04	±	0.93	2.83	±	0.95
Hormonal Contraception	10 (71.43%)			11 (44.00%)			21 (53.85%)		

Note: Continuous variables are presented as mean ± standard deviation; categorical variables are presented as n (%). Heaviness of bleeding was assessed on a five-point Likert scale (1 = very light; 5 = extremely strong). Weekly training duration is represented in hours. Cycle and menstruation length are represented in days. Age and age of menarche are presented in years

### Study design

The study followed a non-randomized controlled design with a no-intervention control group. Randomization was not feasible due to practical constraints, as participant briefings were conducted and study materials were provided in person. Therefore, the EG was recruited locally, while the CG was recruited separately. Two weeks prior to the intervention phase, the participants completed a baseline screening. During the seven-week intervention phase, the EG received an online mindfulness program accessible via the university’s learning platform. The CG continued their usual activities and received no intervention during this period. After the intervention phase, the participants conducted a post-screening that used the same psychometric instruments as the baseline screening. A follow-up screening to assess the maintenance of effects was conducted three months laterand was completed by 17 participants (43.6% of the initial sample), including nine in the EG (64.3%) and eight in the CG (32.0%). ​ As the retained group sizes were considered too small to support reliable between-group analyses, ​follow-up outcomes were not analysed. Following the study, all CG participants were debriefed on the procedures and content and provided access to the intervention materials to complete the program independently. The investigation was conducted in accordance with the Helsinki Declaration on human research, and ethical approval was provided by the local ethics committee (EKS V 2024_21.1).

### Psychometric screening

The baseline and post-screening were conducted via the online platform SoSci Survey. Menstrual symptoms and mental health were assessed at both time points. In addition, demographic and sport-specific characteristics as well as multiple characteristics of menstrual health were assessed at baseline, including age at menarche, cycle length, menstrual length, bleeding heaviness, cycle regularity, intermenstrual bleeding, and hormonal contraception use. Hormonal contraceptive use was assessed as a binary variable (use vs. non-use). Specific contraceptive methods or preparations were not recorded. Post-screening, participants were also asked to share their subjective perceptions of the intervention’s success, including its overall effectiveness, positive and negative effects, impact on menstruation, general feedback on the intervention, and potential future use of mindfulness-based exercises and techniques.

Menstrual symptoms were assessed using the Menstrual Symptom Index (MSi) adapted from Bruinvels et al. [16]. This index comprises 18 symptoms, rated on a four-point Likert scale (0 = *never*; 1 = *rarely*; 2 = *sometimes*; 3 = *often*). Participants were asked how often they experienced each symptom during menstrual bleeding. The total score (range: 0–54) was calculated by summing all item scores. The instrument has been widely used in sports science research in recent years [16, 17, 19, 37, 38]. Mental health was assessed using the 21-item Depression Anxiety Stress Scales (DASS-21) [39, 40], which measures symptoms of depression, anxiety, and stress. Each of the three subscales was evaluated by seven items, rated on a four-point Likert scale ranging from 0 (*did not apply to me at all*) to 3 (*applied to me very much or most of the time*), referring to the past week. According to the authors [40], sum scores above 10 on the Depression and Stress subscales indicate elevated psychopathology, whereas scores above 6 on the Anxiety subscale suggest heightened anxiety symptoms.

### Intervention

The intervention was based on a publicly available online mindfulness program originally developed by a German health insurance provider (Techniker Krankenkasse). The original program was developed by experienced supervisors, based on the MBSR concept. The interdisciplinary team of the Department of Sport Psychology selected and thematically grouped the existing articles, exercises, and audio recordings into seven weekly modules delivered through Moodle. Each module included psychoeducational texts, guided exercises, and brief take-home assignments to be completed throughout the week. The practice schedule was adapted to two assigned exercises on three to four days per week. Participants received a diary to document their experiences and reflections on the exercises. Additionally, participants could mark completed tasks using the Moodle progress tracking function to monitor their own progress throughout the intervention. Adherence was assessed with a single self-report item in the post-intervention assessment (‘How regularly did you complete the exercises and assignments of the intervention’) with five response options (*rather rarely*; l*ess than half of the prescribed amount*; *more than half of the prescribed amount*; *almost as prescribed; exactly as prescribed*). The Moodle progress tracking served participants’ self-monitoring only and was not analysed. The content of the MBSR modules is described in Table 2. The modules were made available online via the university’s Moodle platform sequentially throughout the intervention period and covered the key mindfulness dimensions of acting with awareness, non-judgmental acceptance, and present-moment attention [20]. All participants in the experimental group were provided with the same materials and weekly schedule over the seven-week intervention period.

**Table 2:** Modules of the mindfulness intervention

Week	Theme	Main Components
**1**	Introduction to mindfulness	Psychoeducation ‘Discovering mindfulness‘ Guided sitting meditation Homework: sitting practice and mindful pauses in daily life
**2**	Mindful sensory awareness	Psychoeducation ‘Being at home in the body‘ Guided body scan Homework: body scan and mindful focus on a sensory experience
**3**	Acting with awareness in daily life	Psychoeducation ‘Autopilot vs. mindful living‘ Guided listening practice and three-step breathing space Homework: mindful awareness of daily activities
**4**	Mindful approach to stress	Psychoeducation ‘Meeting stress with mindfulness‘ Guided practice focusing on body, thoughts, feelings, impulses Homework: sitting or walking meditation
**5**	Acceptance of thoughts	Psychoeducation ‘Turning toward experience‘ Guided mountain meditation Homework: sitting meditation and mindful observation of thoughts
**6**	Acceptance of emotions	Psychoeducation ‘Dealing with difficult feelings‘ Guided practice ‘Being with a difficult feeling‘ Homework: unguided practice and allowing emotions consciously
**7**	Self-compassion and kindness	Psychoeducation ‘Self-kindness and self-care‘ Guided three-step self-compassion practice Homework: self-care activity and repeated self-compassion practice

### Statistical analysis

All statistical analyses were conducted using R (version 4.6.1). Linear mixed-effects models were fitted using the *lme4* package to account for the repeated-measures structure of the data, with measurements obtained at pre- and post-intervention. The models included a random intercept for participants to account for the dependency of repeated measurements within individuals. Fixed effects included age, contraceptive use, weekly training duration in hours, MSi score, group (control and experimental), and time point (pre- vs. post-intervention). In addition, all two-way interactions and the three-way interaction among MSi score, group, and measurement time point were included. Age, hormonal contraceptive use, and weekly training duration were selected a priori as potential confounders. The MSi score was entered as a time-varying predictor, using the score obtained at the respective time point. Separate models were fitted for each DASS-21 subscale *Depression*, *Anxiety*, and *Stress*. Mental health scores assessed before and after the intervention were analysed as repeated observations of the respective outcome. Heaviness of bleeding was not included as a covariate in the primary models due to its highly unbalanced distribution, with the majority of participants reporting a medium level (*n* = 24). To assess the robustness of the findings, sensitivity analyses were conducted by additionally adjusting the models for heaviness of bleeding. Continuous predictors, including age, weekly training duration, and MSi score, were grand-mean centered prior to model fitting to facilitate interpretation of the intercept and reduce potential multicollinearity between main effects and interaction terms. Models were estimated using restricted maximum likelihood (REML). Statistical significance of fixed effects was assessed using t-tests with Satterthwaite’s approximation for degrees of freedom. Model assumptions were assessed by visual inspection of Q-Q plots and residual-versus-fitted plots of standardized residuals to evaluate normality and homoscedasticity, respectively. Multicollinearity among fixed effects was assessed using variance inflation factors (VIFs), with values below 5 considered indicative of no problematic multicollinearity. Marginal and conditional R² values were calculated as measures of explained variance, representing the variance explained by the fixed effects alone and by the complete model including both fixed and random effects, respectively. Effect estimates were reported with 95% confidence intervals and *p*-values. Missing data were not imputed. The models included all available observations with complete data on the outcome and all predictors, so that participants with data at only one time point also contributed to the estimation. Estimates are therefore valid under the assumption that data are missing at random. All participants were analysed in the group to which they had been allocated, irrespective of self-reported adherence, and no per-protocol analysis was conducted. Between-group differences at baseline were assessed using Welch’s t-tests or Mann-Whitney U tests for continuous variables, depending on the distribution, and Fisher’s exact test for categorical variables. Normality of continuous variables was assessed using Shapiro-Wilk tests and visual inspection of Q-Q plots. Descriptive data are presented as mean ± SD.

## Results

### Descriptive statistics and baseline comparability

**Fig. 1:**
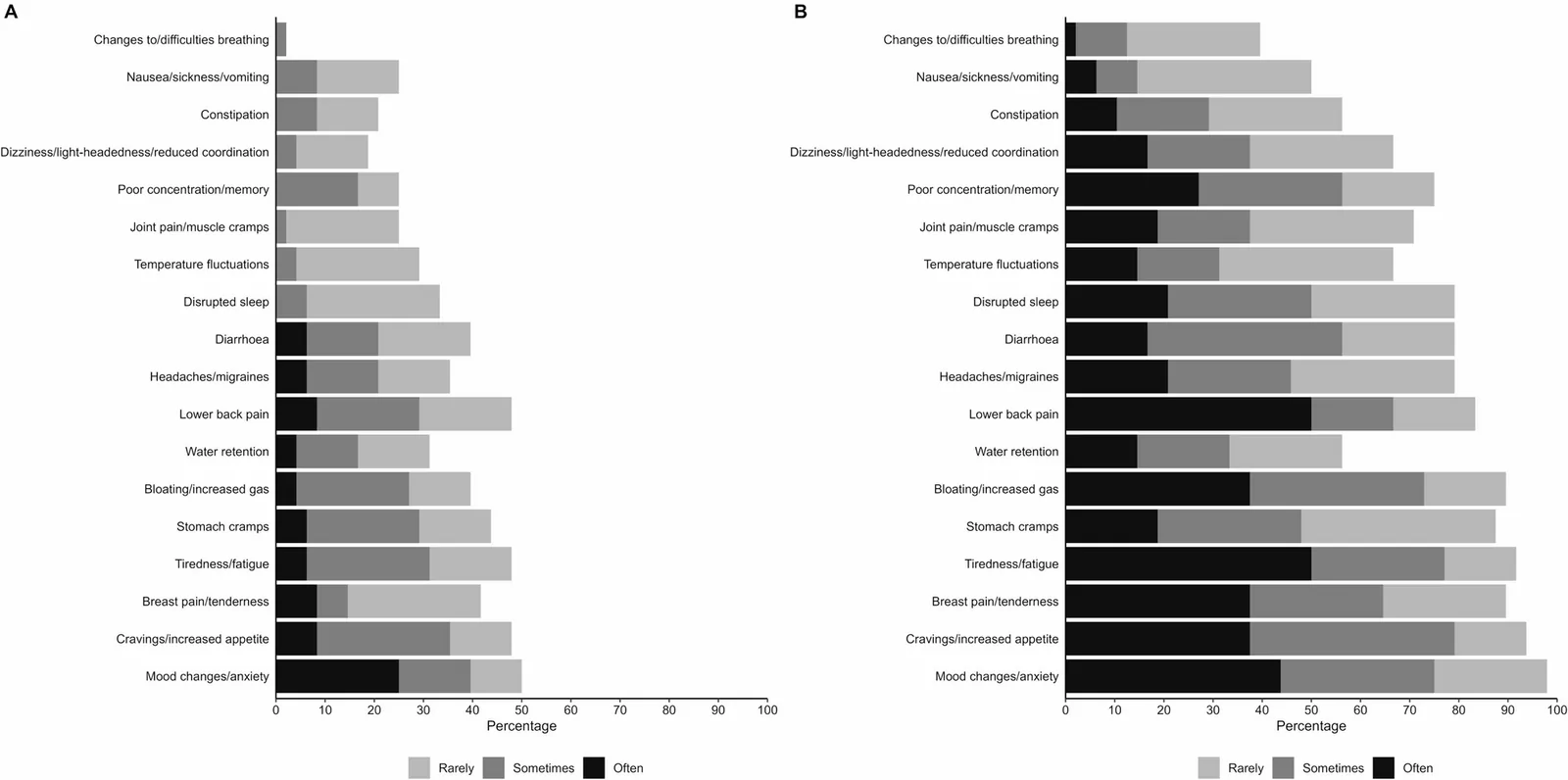
Experienced symptoms by **A**: experimental group (*n* = 14) and **B**: control group (*n* = 25)

Figure 1 illustrates the reported symptom types and their frequency in each group. Descriptively, the control group showed higher total MSi scores than the experimental group, at both pre- and post-measurement (Table 3). At baseline, no statistically significant between-group differences were observed for age, weekly training duration, hormonal contraception, depression, anxiety, or stress (all *p* > .05), whereas the CG showed significantly higher MSi scores than the EG (*t*(35.99) = 2.86; 95% CI [2.66, 15.59]; *p* = .007). The most commonly experienced symptoms included ‘Mood changes / anxiety’, ‘Cravings / increased appetite’, and ‘Lower back pain’.

**Table 3:** Descriptive results for MSi score and DASS-21 scales

	Experimental Group (*n* = 14)						Control Group (*n* = 25)					
	Pre			Post			Pre			Post		
MSi Score	16.71	±	7.09	19.15	±	8.21	25.83	±	12.56	27.83	±	11.73
Depression	5.14	±	3.53	4.15	±	2.23	5.08	±	3.59	6.25	±	4.67
Anxiety	3.64	±	4.05	3.46	±	2.54	4.54	±	3.19	5.92	±	4.04
Stress	5.93	±	3.02	6.08	±	2.22	8.00	±	3.84	8.88	±	4.99

Note. MSi = Menstrual Symptom index. Values are based on all completed assessments at baseline

### Primary analyses

Visual inspection of the diagnostic plots indicated no substantial violations of the model assumptions, although some deviations from normality were observed in the upper tail of the residual distribution for the anxiety model. VIF values were below 5 across all models, indicating no problematic multicollinearity. The post-intervention assessment was completed by 37 participants (EG: *n* = 13; CG: *n* = 24). Of the resulting 76 observations, eleven were not included because of missing values on at least one model variable. Each primary model therefore included 65 observations from 36 participants (EG: *n* = 14; CG: *n* = 22). In the depression model, higher menstrual symptom frequency (MSi) was associated with higher depression scores at baseline in the control group (B = 0.14; 95% CI [0.03, 0.25]; *p* = .012). Neither age, hormonal contraception, weekly training duration, group, nor measurement time point showed significant main effects. Furthermore, none of the interaction terms reached statistical significance, providing no evidence that the relationship between MSi and depression differed across groups or time points.

**Fig. 2:**
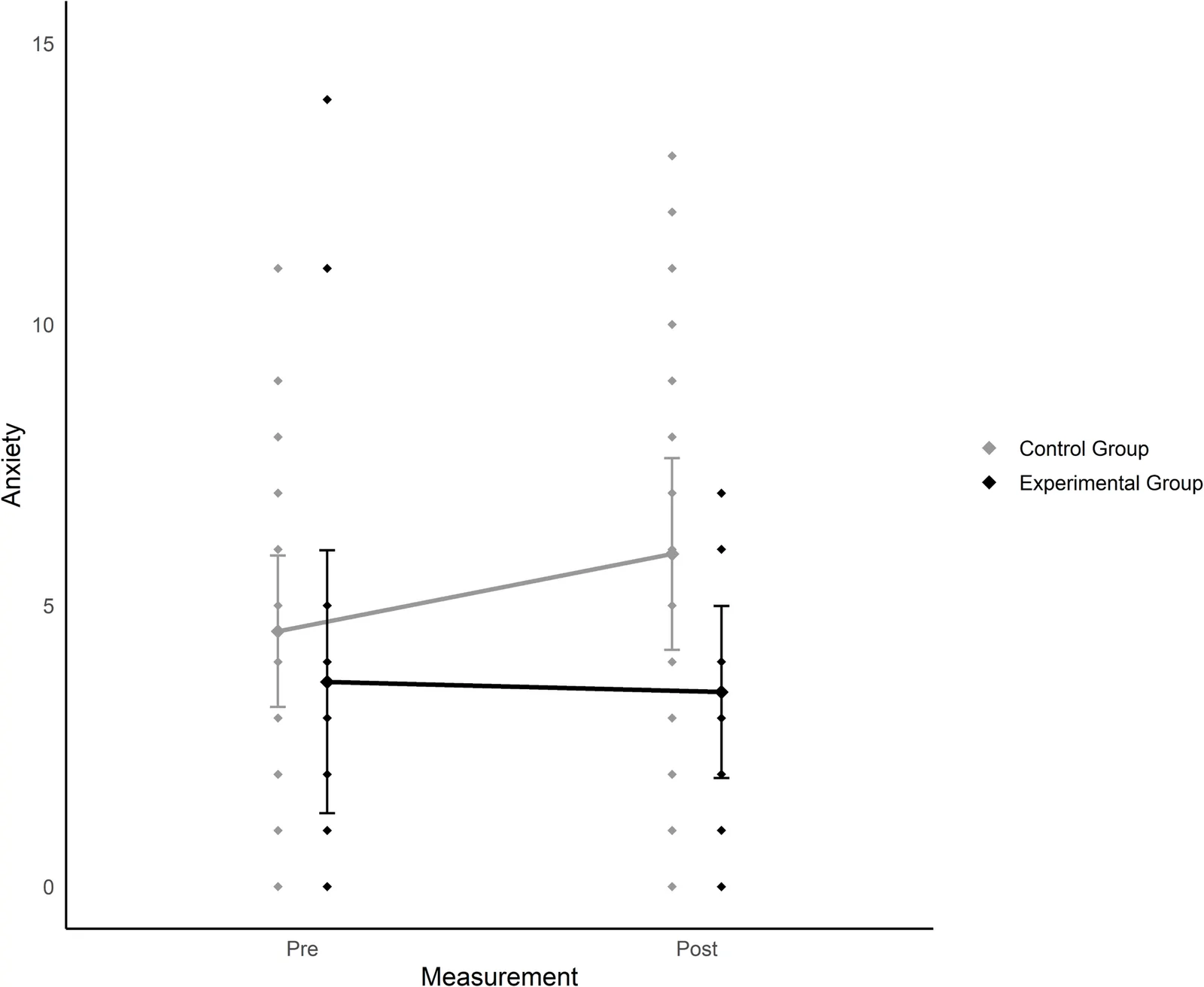
Anxiety scores at pre- and post-measurement in the experimental and control groups. Note Individual observations and group means are displayed as data points; error bars indicate 95% confidence intervals around the group means.

**Fig. 3:**
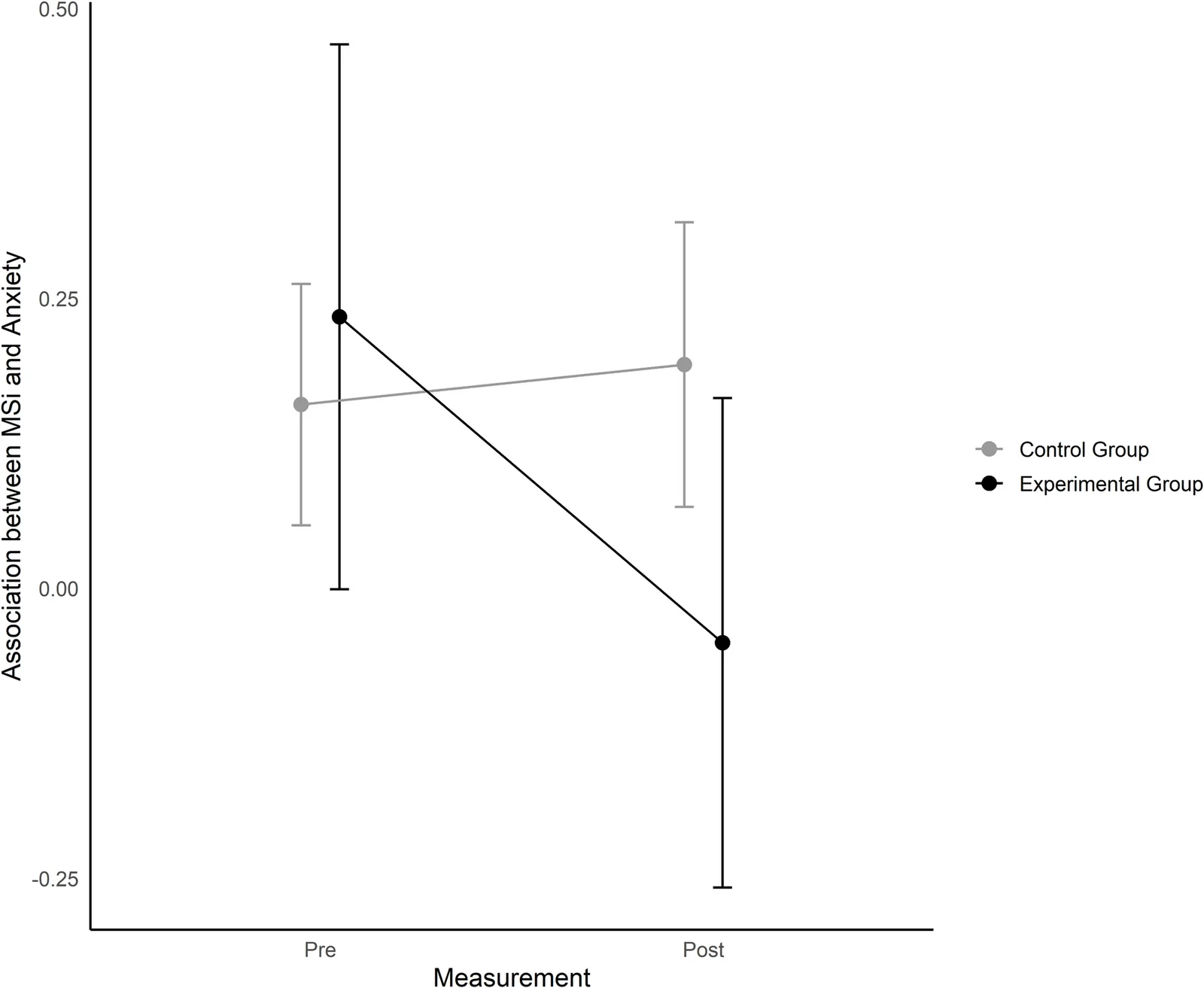
Estimated association between Menstrual Symptom index (MSi) score and anxiety by group and measurement time point. Note Data points represent estimated slopes for the association between MSi score and anxiety; error bars indicate 95% confidence intervals.

In the anxiety model, higher MSi scores were associated with higher anxiety scores at baseline in the CG (B = 0.16; 95% CI [0.05, 0.26]; *p* = .004). At the grand-mean MSi score, changes in anxiety from pre- to post-intervention differed significantly between groups (B = − 3.30; 95% CI [− 6.50, − 0.11]; *p* = .043; Table 4). Descriptively, mean anxiety scores decreased slightly in the EG and increased in the CG (Fig. 2). In contrast, the three-way interaction between MSi, group, and measurement time point did not reach statistical significance (*B* = − 0.32; 95% CI [− 0.64, 0.01]; *p* = .054). Descriptively, the positive association between MSi and anxiety appeared most pronounced in the EG at pre-measurement and weaker after the intervention, whereas it remained relatively stable in the CG (Fig. 3). However, this descriptive pattern should be interpreted cautiously.

**Table 4:** Result of the linear mixed-effects model for the anxiety scale of the DASS-21

Variable	Estimate	SE	95% CI		df	t	*p*
			LL	UL			
Intercept	4.15	0.88	2.37	5.93	41.97	4.71	**< 0.001**
Age^a^	−0.03	0.11	−0.24	0.19	43.33	−0.24	0.813
Hormonal contraception^b^	0.19	0.94	−1.74	2.11	31.05	0.20	0.845
Weekly training duration^a^	−0.20	0.15	−0.51	0.11	26.28	−1.32	0.198
MSi Score^a^	0.16	0.05	0.05	0.26	52.03	3.06	**0.004**
Group^c^	1.28	1.37	−1.47	4.04	49.51	0.94	0.354
Time^d^	1.04	0.83	−0.65	2.74	30.00	1.26	0.218
MSi Score^a^ × Group^c^	0.08	0.13	−0.18	0.33	53.33	0.59	0.557
MSi Score^a^ × Time^d^	0.03	0.07	−0.10	0.17	33.54	0.51	0.615
Group^c^ × Time^d^	−3.30	1.56	−6.50	−0.11	30.43	−2.11	**0.043**
MSi Score^a^ × Group^c^ × Time^d^	−0.32	0.16	−0.64	0.01	37.31	−1.99	0.054

Note. CI = confidence interval; LL = lower limit; UL = upper limit

^a^ continuous variables were grand-mean centered. ^b^ 0 = no hormonal contraception (reference), 1 = hormonal contraception. ^c^ 0 = control group (reference), 1 = experimental group. ^d^ 0 = pre (reference), 1 = post

**Fig. 4:**
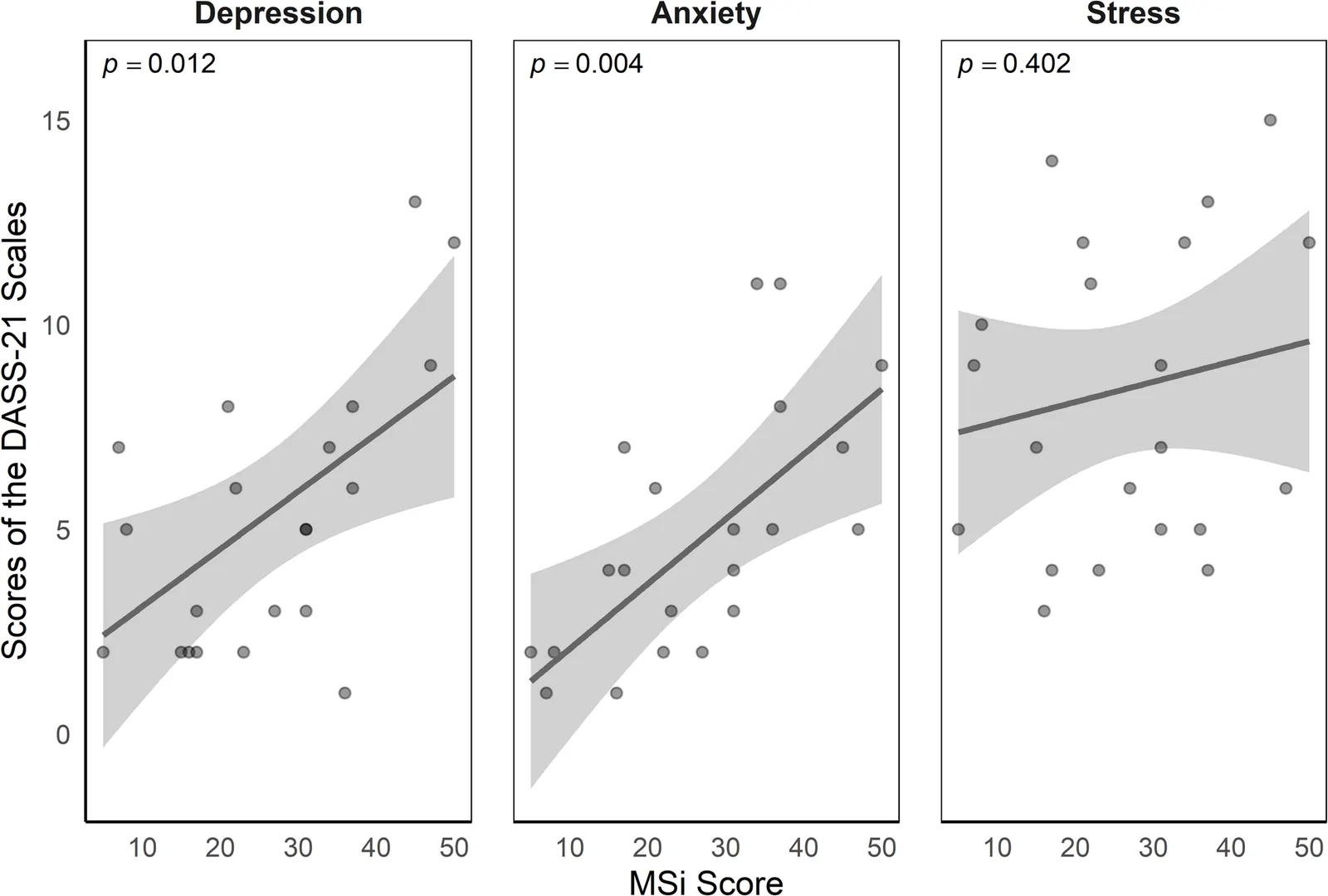
Associations between MSi Score and DASS-21 Scales

Finally, the linear mixed-effects model for stress revealed no statistically significant main effects or interactions. The associations between MSi scores and the DASS-21 scales are displayed in Fig. 4. Sensitivity analyses additionally adjusting for heaviness of bleeding yielded comparable effect estimates and did not materially alter the overall pattern of results. For anxiety, the group × time interaction was not significant in the reduced sample with available data on heaviness of bleeding, irrespective of whether heaviness of bleeding was included in the model. The marginal and conditional R² values were 0.247 and 0.728 for depression, 0.347 and 0.590 for anxiety, and 0.241 and 0.688 for stress, respectively. The tables, including the LMM results for depression and stress, are provided in the Supplementary Material.

### Intervention adherence

Feedback on the intervention was obtained from 13 participants in the EG. Adherence to the prescribed exercises of the MBSR-based intervention was generally high: 46.2% (*n* = 6) of participants practiced almost as prescribed, 15.4% (*n* = 2) exactly as prescribed, and 15.4% (*n* = 2) practiced more than half of the prescribed exercises. However, 23.1% (*n* = 3) reported practicing less than half of the prescribed. Given the small number of participants per adherence category, associations between self-reported adherence and changes in mental health outcomes were not tested inferentially. Descriptive pre-to-post changes in the DASS-21 scales for participants with lower and higher self-reported adherence are provided in the Supplementary Material for exploratory purposes only. In retrospect, 69.2% (*n* = 9) of participants agreed that they had become more mindful in everyday life, whereas 30.8% (*n* = 4) reported neither agreement nor disagreement. Regarding engaging more consciously with mindfulness, 76.9% (*n* = 10) agreed and 7.7% (*n* = 1) strongly agreed, while 15.4% (*n* = 2) neither agreed nor disagreed. Regarding perceived effects, 92.3% (*n* = 12) of participants agreed that the intervention positively affected their well-being, while 7.7% (*n* = 1) reported neither agreement nor disagreement. For perceived effects on sleep, 38.5% (*n* = 5) agreed, whereas 30.8% (*n* = 4) each reported neither agreement nor disagreement or disagreement. Regarding stress, 69.2% (*n* = 9) agreed and 15.4% (*n* = 2) strongly agreed that the intervention had a positive effect. Also, 15.4% (*n* = 2) neither agreed nor disagreed. However, no participant reported a change in their perception of menstruation following the intervention. Finally, concerning future application, 46.2% (*n* = 6) indicated that they would rather apply the techniques in the future, 23.1% (*n* = 3) reported that they would definitely do so, 23.1% (*n* = 3) indicated they would rather not apply them, and 7.7% (*n* = 1) reported that they would definitely not.

## Discussion

The present study examined how menstrual symptom frequency relates to mental health outcomes in physically active female students, and whether these effects could be moderated by an online MBI. Higher MSi scores were associated with higher depression and anxiety scores at baseline in the control group. Changes in anxiety differed between groups at the grand-mean MSi score. However, the MSi × group × time interaction did not reach statistical significance, providing no conclusive evidence of a moderating effect, although the descriptive pattern suggested a weaker association between MSi and anxiety after the intervention. By examining self-reported symptom frequency rather than focusing exclusively on clinically diagnosed menstrual disorders, this study provides novel insights into menstrual symptom frequency and mental health in physically active young women within the context of an online MBI.

### Discussion of results

Previous research has demonstrated positive associations between MSi and mental health outcomes, as measured by the DASS-21, in physically active women and athletes [19]. The present study was partially consistent with these findings, showing significant associations for depression and anxiety, but not with stress. Similarly, Brown et al. [38] observed that athletes experience a range of physical, emotional, and psychological cycle-related symptoms that vary in timing and intensity across the cycle. Notably, the significant group × time interaction for anxiety was reflected in increasing anxiety levels in the control group and decreasing levels in the experimental group. Although MSi scores increased descriptively in both groups, anxiety followed different trajectories. One possible explanation is that emotion regulation processes associated with mindfulness, particularly greater cognitive reappraisal and reduced rumination and worry [34], also shape how menstrual symptoms and accompanying emotional fluctuations are appraised [15, 23]. Nevertheless, this interpretation remains speculative because these mechanisms were not directly assessed, and the non-significant MSi ×group × time interaction provided no statistical evidence that the association between menstrual symptoms and anxiety changed differently between groups over time.

The potential relevance of these processes is underscored by evidence that women with greater difficulties in emotion regulation reported smaller increases in perceived control over anxiety-related events in the late luteal phase [41]. Nonjudgmental awareness and acceptance cultivated through MBSR could help reduce perceived symptom burden by fostering a more balanced self-compassionate attitude toward menstrual experiences. More broadly, the observed group-specific pattern in anxiety is consistent with previous research indicating beneficial effects of MBI on anxiety. Tingaz et al. [32] reported that higher mindfulness was associated with lower depression, anxiety, and stress in student athletes, while Abarkar et al. [42] found that adolescent-oriented mindfulness training significantly reduced social anxiety symptoms in female high-school students. In the present study, intervention-related changes were observed only for anxiety, but not for depression or stress. Differences in study populations, intervention characteristics, and outcome measures may contribute to these divergent findings. Thus, the present findings should not be interpreted as evidence of a broader improvement in mental health.

Given that all participants in the present study were physically active, the sample represents a population already engaging in regular exercise, which has been associated with better menstrual health, including lower levels of pain and emotional distress [12, 43]. However, the experimental group consisted primarily of sport science students, who also reported descriptively higher average training hours per week than the control group, which was mainly composed of psychology students. Baseline comparison demonstrated lower MSi scores in the experimental group, broadly aligning with previous evidence suggesting that regular exercise may alleviate premenstrual symptoms [43], although the present study assessed symptoms during rather than before menstruation. The absence of a significant relationship between weekly training duration and mental health outcomes may reflect that training hours alone do not capture potentially relevant characteristics such as exercise intensity, type, or context. Moreover, training data were collected during screening rather than through daily monitoring and therefore did not capture short-term fluctuations in training load.

Despite the psychological benefits associated with physical activity, regular exercise does not necessarily protect against psychological strain in athletic populations. High physical and psychological demands in competitive and dual-career environments have been associated with greater vulnerability to mental health problems such as anxiety and depression [4, 33]. In addition to academic pressures, student-athletes may face sport-related stressors, including performance expectations, injuries, and selection challenges [5, 6]. These challenges may be particularly relevant for female athletes, who have been reported to experience higher anxiety levels than their male counterparts [2, 44] and may additionally face menstrual cycle-related challenges affecting training and competition [12, 45].

When interpreting menstrual-related mental health outcomes, the potential influence of hormonal contraception must also be considered. Hormonal contraceptive use is common among female athletes, with approximately one in four users reporting cycle regulation and symptom management as primary reasons for use [46]. Although Buggio et al. [47] suggest that hormonal contraception may be associated with depressive symptoms, particularly among adolescents, the overall evidence remains inconsistent and does not provide conclusive support for a causal relationship. In the present study, 53.85% (*n* = 21) of the participants reported using hormonal contraceptives. However, the use of hormonal contraception was not significantly associated with mental health outcomes, which aligns with previous findings reporting no differences in psychological well-being or symptom occurrence between users and non-users [19, 37, 45, 48]. Different hormonal contraceptive preparations may have different effects on menstrual symptoms and mental health. These differences were beyond the scope of the present study and could not be fully accounted for by the binary assessment of contraceptive use. Additional subgroup analyses by hormonal contraceptive use were not conducted because the resulting subgroups were small, with only four non-users in the EG, limiting the precision of subgroup-specific estimates. Further research considering specific types of hormonal contraception may help clarify these associations. In this context, MBI may represent a complementary non-pharmacological approach for supporting menstrual-related well-being and mental health.

Adherence to the MBI in this study was generally high, with most participants (77%; *n* = 10) reporting that they had practiced at least half of the prescribed exercises and describing positive effects on well-being, stress, and everyday mindfulness. This aligns with Gordon et al. [49], who found that greater engagement in MBSR home practice was associated with lower depressive symptoms in perimenopausal women, suggesting that the extent of practice may be relevant to intervention outcomes. Moreover, the high self-reported adherence and positive feedback suggest that a self-guided online format may provide a low-threshold and flexible approach to deliver MBSR among young women. Furthermore, Askari et al. [29] reported sustained improvements at a one-month follow-up. However,​the retained group sizes were considered too small for reliable between-group analyses of follow-up outcomes. Future research should ensure adequate sample sizes to enable reliable follow-up assessments.

### Limitations

Several limitations of the present study should be acknowledged. First of all, the intervention was not specifically designed for women or tailored to the academic or sporting context. Considering that coping strategies and stress experiences are strongly shaped by gender, sport type, and cultural environment [50], future MBI should be developed with an explicit focus on menstrual symptomatology and adapted to academic demands and the specific demands of different sports, as each discipline carries distinct psychological and physiological risk factors [51]. Considering the generalization, methodological constraints limit the findings. The relatively small and unbalanced group sizes (EG = 14, CG = 25) may have reduced statistical power, particularly for detecting interaction effects. In addition, the small retained samples at follow-up and the unequal retention rates between groups limited our ability to evaluate long-term effects. Furthermore, the groups differed significantly in baseline MSi scores, with higher scores in the CG. Although MSi was accounted for in the LMM, this baseline imbalance should be considered when interpreting the findings. Moreover, the absence of an active control group and the non-randomized study design limit causal interpretation of the observed group differences. The different recruitment sources may have introduced selection bias and contributed to pre-existing differences between groups, which should be considered when interpreting the findings. Although age, hormonal contraceptive use, and weekly training duration were included as covariates, residual confounding by unmeasured factors such as academic workload or expectations regarding the intervention cannot be ruled out, particularly as the CG received no intervention. Moreover, menstrual symptoms and mood may fluctuate over time independently of any intervention. With only two measurement points, intervention-associated changes cannot be separated from such natural variation or from regression to the mean, especially given the baseline difference in MSi scores.

As intervention adherence was assessed retrospectively by self-report, the accuracy of adherence estimates may have been limited. The descriptive changes by adherence level reported in the Supplementary Material are based on very small subgroups and do not permit conclusions about a dose-response relationship. Future studies should therefore monitor adherence objectively and examine it as a predictor of outcomes to provide more nuanced insights on whether differences in engagement are associated with differential responses to MBI. Finally, the assessment relied on self-reported data, which are susceptible to recall and response bias, particularly when reflecting on symptoms retrospectively rather than monitoring them daily or at least weekly. As menstrual symptoms and mental health were assessed concurrently and referred to different time frames, the observed associations do not allow conclusions about temporal order or direction. Prospectively, adequately powered randomized controlled designs should incorporate longer follow-up periods and more frequent measurements or continuous symptom monitoring to obtain a more differentiated picture of symptom burden, for example, by combining symptom frequency and severity (e.g., MSi × severity score) [37]. Furthermore, future research should investigate whether the effectiveness of MBI differs depending on baseline menstrual symptom burden. Although MBSR interventions are generally regarded as safe, mindfulness practice may temporarily heighten negative emotions or worries in some individuals. Thus, careful participant screening and ongoing guidance are recommended to ensure psychological safety and maximize intervention effectiveness. Another limitation is the retrospective registration of the study. As trial registration was not considered at study initiation, the study was registered retrospectively after completion of data collection. No protocol elements were prospectively registered, which limits transparency regarding selective reporting. Prospective registration would have provided greater transparency.

## Conclusion

The present study revealed that higher menstrual symptom frequency was associated with higher anxiety and depression scores at baseline in the control group. At the grand-mean MSi score, changes in anxiety over time differed between the intervention and control groups. However, the three-way interaction between MSi, group, and time did not reach statistical significance. Given the limited sample size and non-randomized design, these findings should be considered preliminary and interpreted with caution. Nevertheless, they suggest that mindfulness may represent a low-threshold, non-pharmacological approach to support menstrual-related well-being. The online, self-guided format showed good adherence and acceptability, suggesting that a digital approach may increase accessibility, especially for students balancing academic and athletic demands. Overall, the present study contributes to the growing understanding of the interplay between menstrual and mental health in physically active women and provides preliminary insights into the potential role of mindfulness in this context. Future research should expand on these findings by employing randomized controlled trials with active control groups, larger samples, daily symptom tracking, and long-term follow-up assessments and by investigating mindfulness programs specifically tailored to menstrual-related concerns and the needs of female students and athletes. If supported by further evidence, MBI may represent a useful component of health promotion programs for women experiencing menstrual-related physical and emotional symptoms.

## Supplementary Information

Below is the link to the electronic supplementary material.

Supplementary Material 1

## Data Availability

The datasets used and/or analysed during the current study are available from the corresponding author on reasonable request.
