# Supplementary material for: The effect of a seven-week online mindfulness intervention on the relationship between menstrual symptoms and mental health - A non-randomized controlled trial
Source: BMC Psychol. 2026 Nov 4;14:1594. doi: 10.1186/s40359-026-05888-2 (PMC13732874; doi:10.1186/s40359-026-05888-2)
Supplement: Supplementary file 1 [file 40359_2026_5888_MOESM1_ESM.docx]

# Depression

| Variable | Estimate | SE | 95% CI | | df | t | *p* |
| --- | --- | --- | --- | --- | --- | --- | --- |
|  |  |  | *LL* | *UL* |  |  |  |
| Intercept | 5.16 | 0.97 | 3.20 | 7.12 | 40.14 | 5.32 | **< .001** |
| Age^a^ | −0.00 | 0.12 | −0.24 | 0.23 | 38.33 | −0.01 | .990 |
| Hormonal contraception^b^ | −0.28 | 1.07 | −2.45 | 1.90 | 37.17 | −0.26 | .798 |
| Weekly training duration^a^ | 0.28 | 0.18 | −0.09 | 0.64 | 29.11 | 1.54 | .133 |
| MSi Score^a^ | 0.14 | 0.05 | 0.03 | 0.25 | 51.28 | 2.62 | **.012** |
| Group^c^ | 0.41 | 1.46 | −2.52 | 3.34 | 46.02 | 0.28 | .779 |
| Time^d^ | 0.79 | 0.71 | −0.66 | 2.24 | 29.27 | 1.12 | .273 |
| MSi Score^a^ x Group^c^ | −0.04 | 0.13 | −0.30 | 0.22 | 53.97 | −0.33 | .742 |
| MSi Score^a^ x Time^d^ | −0.01 | 0.06 | −0.13 | 0.11 | 31.53 | −0.21 | .838 |
| Group^c^ x Time^d^ | −2.19 | 1.34 | −4.92 | 0.55 | 29.95 | −1.63 | **.**113 |
| MSi Score^a^ x Group^c^ x Time^d^ | 0.03 | 0.14 | −0.26 | 0.31 | 34.58 | 0.19 | .850 |

*Note.* CI = confidence interval; LL = lower limit; UL = upper limit.

^a^ continuous variables were grand-mean centered. ^b^ 0 = no hormonal contraception (reference), 1 = hormonal contraception. ^c^ 0 = control group (reference), 1 = experimental group. ^d^ 0 = pre (reference), 1 = post.

# Stress

| Variable | Estimate | SE | 95% CI | | df | t | *p* |
| --- | --- | --- | --- | --- | --- | --- | --- |
|  |  |  | *LL* | *UL* |  |  |  |
| Intercept | 8.55 | 1.04 | 6.45 | 10.66 | 41.00 | 8.20 | **< .001** |
| Age^a^ | −0.06 | 0.13 | −0.31 | 0.19 | 39.95 | −0.48 | .632 |
| Hormonal contraception^b^ | −0.54 | 1.15 | −2.87 | 1.78 | 36.46 | −0.47 | .638 |
| Weekly training duration^a^ | 0.26 | 0.19 | −0.13 | 0.65 | 29.43 | 1.38 | .178 |
| MSi Score^a^ | 0.05 | 0.06 | −0.07 | 0.17 | 51.44 | 0.84 | .402 |
| Group^c^ | −1.67 | 1.58 | −4.84 | 1.50 | 47.04 | −1.06 | .294 |
| Time^d^ | 0.42 | 0.81 | −1.23 | 2.06 | 30.42 | 0.52 | .610 |
| MSi Score^a^ x Group^c^ | 0.11 | 0.14 | −0.17 | 0.40 | 53.85 | 0.79 | .435 |
| MSi Score^a^ x Time^d^ | 0.05 | 0.07 | −0.09 | 0.19 | 32.90 | 0.74 | .462 |
| Group^c^ x Time^d^ | −0.98 | 1.53 | −4.09 | 2.14 | 30.99 | −0.64 | .527 |
| MSi Score^a^ x Group^c^ x Time^d^ | −0.04 | 0.16 | −0.36 | 0.28 | 35.97 | −0.23 | .816 |

*Note.* CI = confidence interval; LL = lower limit; UL = upper limit.

^a^ continuous variables were grand-mean centered. ^b^ 0 = no hormonal contraception (reference), 1 = hormonal contraception. ^c^ 0 = control group (reference), 1 = experimental group. ^d^ 0 = pre (reference), 1 = post.

# Exploratory descriptive changes in DASS-21 scores by self-reported adherence (experimental group)

| Self-reported adherence | *n* | Depression | Anxiety | Stress |
| --- | --- | --- | --- | --- |
| Lower (less or more than half of the prescribed amount) | 3 | −1.67 (3.06) | 1.00 (2.65) | 1.00 (2.00) |
| Higher (almost or exactly as prescribed) | 8 | −1.63 (2.45) | −0.38 (3.11) | −1.38 (2.39) |

Note. Values are mean (SD) pre-to-post change scores (post minus pre); negative values indicate a decrease. Adherence categories were combined post hoc because of small group sizes. Change scores were available for 11 of the 13 participants who provided adherence ratings. No inferential tests were conducted.
